# SHH induces macrophage oxidative phosphorylation and efferocytosis to promote scar formation

**DOI:** 10.1186/s12964-024-01692-w

**Published:** 2024-06-19

**Authors:** Julei Zhang, Zeliang He, Chenlu Xiong, Yuanyuan Yao, Chengliang Zhang, Wende Yao, Sihan Yang, Xiaodong Li, Yan Han

**Affiliations:** 1 Department of Plastic and Reconstructive Surgery, The First Medical Centre, Chinese PLA General Hospital, 28 Fuxing Street, Beijing, 100853 China; 2 Department of Burn and Plastic Surgery, The 980st Hospital of the PLA Joint Logistics Support Force, 398 Zhongshan Road West, Shijiazhuang, Hebei China; 3 School of Medicine, Nankai University, Tianjin, China; 4 Department of Plastic Surgery, Shaoxing People’s Hospital, Shaoxing, Zhejiang China; 5 Department of Plastic Surgery, Peking University International Hospital, Beijing, China; 6 Department of Plastic Surgery, The First Affiliated Hospital of Anhui Medical University, Hefei, Anhui China

**Keywords:** Sonic hedgehog, Scar formation, Vismodegib, Fibrosis, Macrophage

## Abstract

Excessive scar formation such as hypertrophic scars and keloids, resulting from trauma or surgical procedures, present a widespread concern for causing disfigurement, discomfort, and functional limitations. Macrophages play pivotal roles in maintaining tissue homeostasis, orchestrating tissue development, repair, and immune responses, and its transition of function and phenotype plays a critical role in regulating the balance between inflammation and tissue regeneration, which is central to cutaneous scar formation. Recent evidence suggests the involvement of Sonic Hedgehog (SHH) in the induction of anti-inflammatory M2-like macrophage phenotypes within tumor microenvironments. In our study, we observed increased SHH expression in human hypertrophic scars, prompting an investigation into its influence on macrophage polarization, efferocytosis, and cutaneous scar formation. Our findings reveal that SHH can enhance oxidative phosphorylation (OXPHOS) in macrophages, augment macrophage efferocytosis, and promote M2 polarization, finally contributing to the progression of cutaneous scar formation. Notably, targeting SHH signaling with vismodegib exhibited promising potential in mitigating scar formation by reversing the effects of enhanced OXPHOS and M2 polarization in macrophages. In conclusion, this study underscores the critical roles of macrophage metabolism, particularly OXPHOS, efferocytosis and SHH signaling in cutaneous scar formation. Understanding these mechanisms provides new avenues for potential interventions and scar prevention strategies.

**Supplementary Information:**

The online version contains supplementary material available at 10.1186/s12964-024-01692-w.

## Introduction

Millions of patients suffer from cutaneous scars every year, as a result of trauma or surgery [1]. Scar formation is a necessary procedure of skin wound healing, but excessive or aberrant scar will lead to disfigurement and disability [2]. Pathological scar such as hypertrophic scar or keloids may develop as a result of delayed wound healing, injury or burn of deep dermal, or injury in the chest, ear lobe and other sensitive regions. At the pathological level, it is mainly featured by excessive proliferation of fibroblasts, massive deposition of ECM (mostly collagen), reduced tensile strength and elasticity, and a lack of hair follicles [3]. Despite not being fatal, hypertrophic scar and keloids may cause cosmetic issues. Besides, they can cause pain or itching. If they form over a joint, they can limit joint movement, and even restrict the growth of trunk and limbs of children [2]. Hypertrophic scar is currently one of the most frequent diseases in plastic surgery. Doctors take several methods to prevent and treat hypertrophic, including local tension controlling during and after suture, pressure treatment, silicone gel, laser therapy, etc. However, owing to the insufficient understanding of the mechanism, the outcome of scar treatment is still unsatisfactory.

Although perfect healing of a deep dermal injury or surgical incision remains impossible, the key period of scar formation has been elucidated. At the late stage of tissue repair, macrophages can, under the stimulation of a number of factors, switch from the classically activated or M1 type to the alternatively activated or M2 type and exert their anti-inflammatory and pro-repair effects by secreting growth factors such as TGF-β [4]. Normally, the conversion of macrophage phenotype and function determines the correct initiation and regression of the local inflammation and proliferation period [5]. However, under pathological conditions, macrophages exhibit enhanced M2 polarization and exert their pro-fibrotic role, leading to excessive scar formation [6], but the underlying reason is still unclear. A key process regulating macrophage polarization is efferocytosis. Through efferocytosis, macrophages first complete the rapid clearance of apoptotic cells and reduce inflammatory stimulation; in addition, macrophages undergoing phagocytosis can initiate the transcriptional translation of M2-related genes and release anti-inflammatory mediators through the regulation of receptor signalling as well as metabolites to complete phenotypic conversion, promote inflammation resolution and initiate tissue repair [7].

SHH is a member of the Hedgehog (Sonic, Indian and Desert hedgehog) family. It is a secreted protein that binds to its receptor Patched on the surface of target cells and activates the downstream transcription factor Gli [8]. SHH is involved in a wide range of developmental processes and tissue patterning during embryogenesis. It has also been shown to regulate cell proliferation, differentiation and migration during the healing process in various tissues [8, 9]. In this study, we found that SHH expression is increased in human hypertrophic scar, and investigated its role in enhancing efferocytosis and M2 polarization of macrophages, thus aggravating cutaneous scar formation. We also investigated a potential strategy of targeting SHH to prevent scar formation in a mouse model of skin wound healing.

## Materials and methods

### Patients’ sample collection

The hypertrophic scar and adjacent normal skin tissue used in this study were collected from 11 patients who underwent plastic surgery at the Department of Burn and Plastic Surgery, 980th Hospital, PLA Joint Logistics Support Force. Patients between the ages of 18 and 65 years with a diagnosis of hypertrophic scars were included. The diagnosis was made by a professional plastic surgeon on the basis of clinical presentation. Characteristics of hypertrophic scars include a clear history of skin injury such as trauma, burns, surgery or injury, a scar that grows above the surrounding skin without extending beyond the original wound edges, a typical red or purple appearance, a hard texture, and symptoms of itching or pain. Patients were excluded if they had been diagnosed with keloids or other systemic diseases, or if they had received scar treatment in the previous 6 months, including hormone injections, radiotherapy, surgery or any other method. Characteristics of keloids include scar tissue that extends beyond the boundaries of the original wound, invading the surrounding normal skin, commonly found on the ears, chest, shoulders or neck, and receding over time. Prior to surgery, all patients signed an informed consent form and all protocols were approved by the Ethics Committee of Bethune International Peace Hospital in accordance with the tenets of the Declaration of Helsinki.

### Primary fibroblast culture from normal skin, hypertrophic scar and mice skin

Human hypertrophic scar, normal skin tissues and mice skin tissues were placed in 0.25% DISPASE enzyme (No.17105041, Gibco, USA) overnight at 4 °C to separate epidermis and dermis. The dermal portion of the skin was cut into tissue blocks less than 1 mm [3], and fibroblasts were isolated by culturing the tissue block explants. Fibroblasts were cultured in Dulbecco’s Modified Eagle Medium (DMEM) (No.C11885500BT, Gibco, USA) supplemented with 10% fetal bovine serum (FBS) (No.10099141 C, Gibco, USA) and 1% penicillin/streptomycin (No.15070063, Gibco, USA). The medium was changed every 2 days. Fibroblasts of the third and fifth sub-passages were used for the following experiments.

### Isolation and induction of bone marrow derived macrophage

Femur and tibia of C57BL/6 mice were collected and bone marrow cells were isolated by cell strainer and centrifugation, and erythrocytes were lysed via hypotonic shock in sterile distilled water. Bone marrow cells were cultured in RPMI 1640 (No.22400089, Gibco, USA) supplemented with 10% FBS and 1% penicillin/streptomycin. M-CSF (30ng/ml, No.574814, Biolegend, USA) was added to the medium to induce macrophage differentiation. 100ng/ml rmSHH was added to the medium for SHH stimulation.

### ELISA

The tissues and the culture supernatant of fibroblasts as described above were collected to assess the concentration of SHH using an ELISA test kit (NO.IPD11539H, IPODIX, China). Mouse COL1 ELISA Kit (No.D721060, Sangon Biotech, China) was used for COL1 determination of the wound area. EZElisa™ Human Collagen Type I ELISA Kit (NO.A-QEK01782-96wells, Biogradetech, USA) and EZElisa™ Human Collagen Type III (COL3) ELISA Kit (NO.A-QEK01779-96wells, Biogradetech, USA) were used to assess the COL1 and COL3 from the supernatant of fibroblasts. TNF-α ELISA Kit (No.EK282, MultiSciences, China) and TGF-β ELISA Kit (No.EK981, MultiSciences, China) were used to assess the TGF-β and TNF-α of macrophage. All procedures were performed according to the manufacturer’s instructions.

### Immunohistochemistry

Tissue samples were fixed in 4% paraformaldehyde, dehydrated in graded ethanol, embedded in paraffin and sectioned for immunohistochemistry. Paraffin sections were incubated with antibodies against SHH (No.ab53281, Abcam, United Kingdom). Images of immunohistochemistry staining were captured with an Olympus microscope, and the positive cell or area was quantified using ImageJ software. Six random fields from every sample were evaluated.

### Real-time quantitative PCR

RNA was extracted from cells using TRIzol Reagent (No.15596026CN, Invitrogen, USA) and its concentration was confirmed quantitatively. RNA was reversely transcribed into cDNA using the Prime Script™ RT Reagent Kit (No. RR037A, Takara, Japan) according to the manufacturer’s instructions. Real-time PCR reaction was performed in SYBR Premix Ex Taq II (No.RR820A, Takara, Japan) with the following PCR cycles: 40 cycles at 95°C for 5 s and at 60°C for 30 s. The primers used in our study were as follows: *GAPDH*, 5’- GGAGCGAGATCCCTCCAAAAT-3’ (forward) and 5’- GGCTGTTGTCATACTTCTCATGG-3’ (reverse); *SHH*, 5’- CTCGCTGCTGGTATGCTCG-3’ (forward) and 5’- ATCGCTCGGAGTTTCTGGAGA-3’ (reverse); *GLI1*, 5’- AGCGTGAGCCTGAATCTGTG-3’ (forward) and 5’- CAGCATGTACTGGGCTTTGAA-3’ (reverse).

### Mice and cutaneous wound model

C57BL/6 mice were selected to establish a mouse cutaneous wound model. After anesthesia by intraperitoneal injection of 1% sodium pentobarbital (40 mg/kg), the dorsal hair was shaved and the skin was prepared according to standard sterile procedures. A 6 mm diameter full-thickness cutaneous wound was established on the back of the mice. For drug administration, 100 µl (1 mg/ml) rm-SHH (No.315 − 22, Peprotech, USA) or 100ul vismodegib (20 mg/kg, No.S1082, Selleck, USA) were subcutaneously injected around the wound site immediately after the surgery and every 24 h post-surgery until the endpoint of the observation. The mice were sacrificed at the 3rd day for the observation of inflammation cells, and 14th day for the evaluation of Masson staining, COL1A1 and CD31. The wounds area was measured with ImageJ software. The healing speed was evaluated by the ratio of unhealed wound to initial wound area.

### Masson staining

Tissue samples from mouse cutaneous wound were made into paraffin sections and Masson staining was performed according to the manufacturer’s instructions provided in the Masson kit (No.D026-1-1, Nanjing Jiancheng, China) to detect the collagen deposition. The thickness of the dermis was measured with ImageJ software.

### Immunofluorescence

After the cultured fibroblasts reached 70–80% confluence, the cells were fixed in 4% paraformaldehyde for 15 min. Paraffin sections of tissue samples were deparaffinized and rehydrated. Both the cell and tissue samples were then washed, permeabilized, and blocked. The following primary antibodies were used: anti-COL1A1 (No.27,026, CST, USA), anti-CD31 (No.GB12063-100, Seivice Bio, China), anti-Ly6G (No.GB11229-100, Seivice Bio, China), anti-F4/80 (No.GB113373-100, Seivice Bio, China), anti-CD206 (No.GB113497-100, Seivice Bio, China), anti-iNOS (No.GB123965-100, Seivice Bio, China). Images of the IF staining were captured with a fluorescence microscope (NIKON, Japan) for the analysis of the fluorescent intensity.

### Mitochondria superoxide detection

To detect the level of superoxide in mitochondria, MitoSox^™^ (No.M36006, Invitrogen, USA) was used according to the manufacturer’s instruction. Briefly, macrophage was cultured in plates and stimulated with rmSHH. Then the MitoSox^™^ Green reagent was applied to cover cells for 30 min incubation at 37 °C and 5% CO_2_. Then the cells were washed gently for 3 times with phosphate buffered saline (PBS). Then the cells were observed and captured with a fluorescence microscope (NIKON, Japan) for the analysis of the fluorescent intensity.

### Flow cytometry

The expression of cell surface markers and the proportion of macrophage phenotype were measured by flow cytometry. Cell and tissue samples were treated with anti-mouse CD16/CD32 (No.553,141, BD Pharmingen, USA) to block cell surface Fc receptors and then incubated with FITC-conjugated anti-F4/80 (No.157309, Biolegend, USA), PE-conjugated anti-CD86 (No.553,692, BD Pharmingen, USA), APC-conjugated anti-CD206 (No.141,707, Biolegend, USA), PerCP-Cy5.5-conjugated anti- CD11b (No.550,993, BD Pharmingen, USA) in the dark. The cells were then analyzed by flow cytometry using BD FACSAria (BD Biosciences, USA). M1 macrophages were defined as F4/80 and CD86. M2 macrophages were defined as F4/80 and CD206.

### CCK8 assay

Fibroblasts from hypertrophic scar were seeded into 96-well plates at a density of 1 × 10^4^ cells per well. The control group was cultured with DMEM and the SHH-stimulated group was cultured with DMEM supplemented with SHH (200pg/mL, No.100 − 45, Peprotech, USA). Cell proliferation was examined using CCK-8 assay at the time points of 24, 48 and 72 h. 10 µL CCK-8 solution (No.C0037, Beyotime, China) was added to each well and cells were incubated at 37 °C for 2 h. The absorbance was detected at 450 nm using a microplate reader.

### Apoptosis analysis: PI/Annexin5

Cell apoptosis was measured with the Annexin V Apoptosis Detection Kit (No.88-8102-72, eBioscience, USA) following the manufacturer’s instructions. 100 µL cell suspension was stained with 10 µL Annexin V-FITC and 5 µL propidium iodide (PI). FITC and PI signals were detected at 515 nm and 620 nm activated by the wave at 488 nm, and the proportion of PI/Annexin V-stained cells was quantified by flow cytometry.

### Efferocytosis induction

Apoptotic neutrophils were incubated with PKH26 (No.PKH26PCL, Sigma, USA) for 30 min to label the cell membrane, followed by coculturing with bone marrow derived macrophage at a ratio of 5:1 for 30 min, and the plates were washed three times with PBS to remove unbound neutrophils. The macrophage phagocytizing apoptotic neutrophils was identified by immunofluorescence and flow cytometry.

### Seahorse Cell Mito Stress Test and ATP production rate

Oxygen consumption rate (OCR) was measured using an Agilent Seahorse XFe24 Analyzer and Cell Mito Stress Test Kit (Agilent Technologies, Cheadle, United Kingdom) according to the manufacturer’s instructions. Cells were seeded into the XFe24 Cell Culture Microplates at 2 × 10^4^ cells/well and incubated for 1 h, then consecutively treated with 1µM oligomycin, 1 µM carbonyl cyanide-p-trifluoromethoxyphenylhydrazone (FCCP), and 1 µM Rotenone/Antimycin A (AA), while OCR is monitored in real-time. Data analysis was conducted using Wave Software (Agilent Technologies, Cheadle, United Kingdom).

### Mitochondrial complex I and complex II activity

Cells were harvested to extract mitochondrial proteins and the activity of mitochondrial complex I and complex II were measured by Complex I Enzyme Activity Microplate Assay Kit (ab109721, Abcam, United Kingdom) and Complex II Enzyme Activity Microplate Assay Kit (ab109908, Abcam, United Kingdom), respectively. Briefly, mitochondrial proteins were added to the 96-well plate, which had been pre-coated with a specific capture antibody, and the complex activity was determined by tracking the oxidation and reduction of the complex-specific substrate. Absorbance was measured using a spectrophotometer at 450 nm (complex I) and 600 nm (complex II), respectively.

### Statistics analysis

Data were analyzed with SPSS 26.0. For comparing data from two groups, student’s *t* test was used. For comparing data from multiple groups, One-way ANOVA was used. *P* < 0.05 was considered statistically significant.

## Results

### SHH expression was increased in hypertrophic scar and fibroblasts

**Fig. 1:**
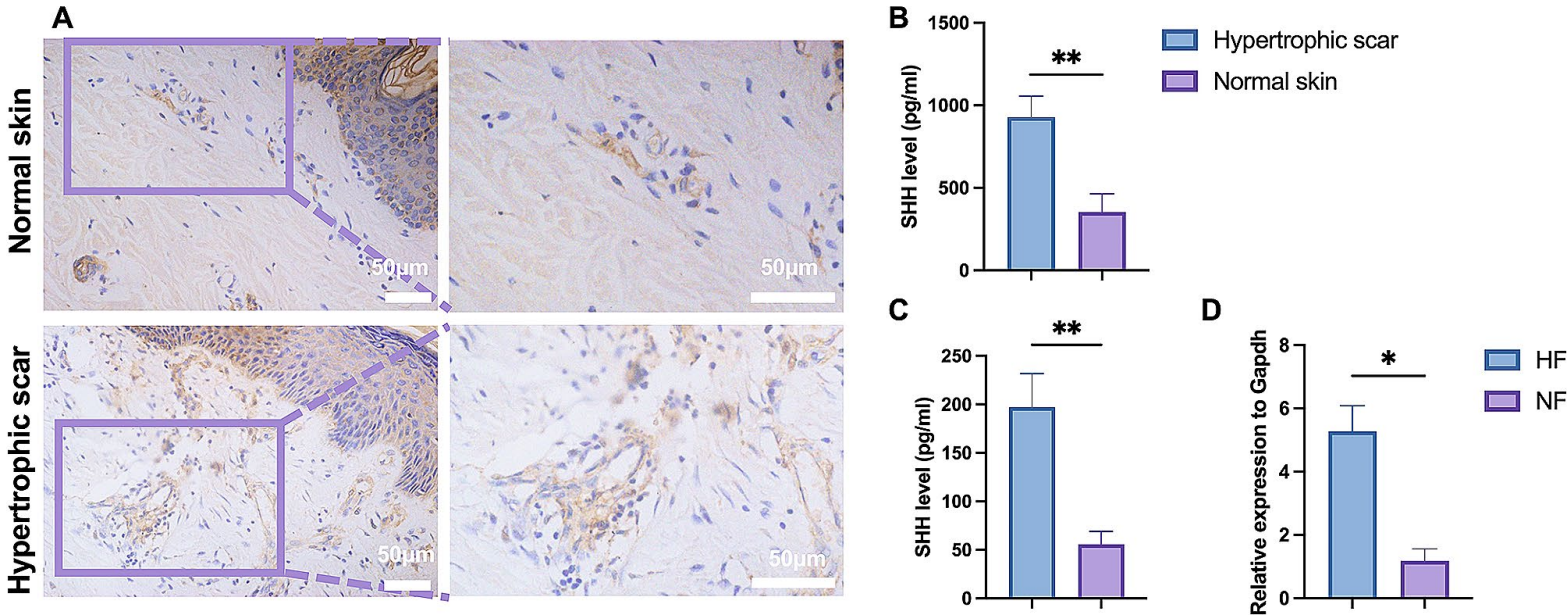
SHH expression was increased in hypertrophic scar and fibroblasts. **A-B**, IHC staining (**A**) and ELISA (**B**) were performed to estimate the expression of SHH in hypertrophic scar and normal skin (*n* = 11). **C-D**, the fibroblasts from hypertrophic scar (HF) and normal skin (NF) was isolated and cultured, and ELISA (**C**) and RT-qPCR (**D**) were performed to evaluate the expression of SHH in HF and NF (*n* = 6). * *p* < 0.05, ** *p* < 0.01

To investigate the expression of SHH in scar tissue, we collected tissues of hypertrophic scar and adjacent normal skin (Supplementary Table 1). IHC and ELISA exhibited that in hypertrophic scar tissue, SHH expression was significantly increased compared with adjacent normal skin (Fig. 1A, B). According to the localization of SHH in IHC, we presumed that SHH was mainly expressed in fibroblasts. Then, we verified the over-expression of SHH in fibroblasts derived from hypertrophic scar tissues than fibroblasts from normal skin tissues (Fig. 1C, D).

### SHH promoted collagen deposition and polarization of M2 macrophage

**Fig. 2:**
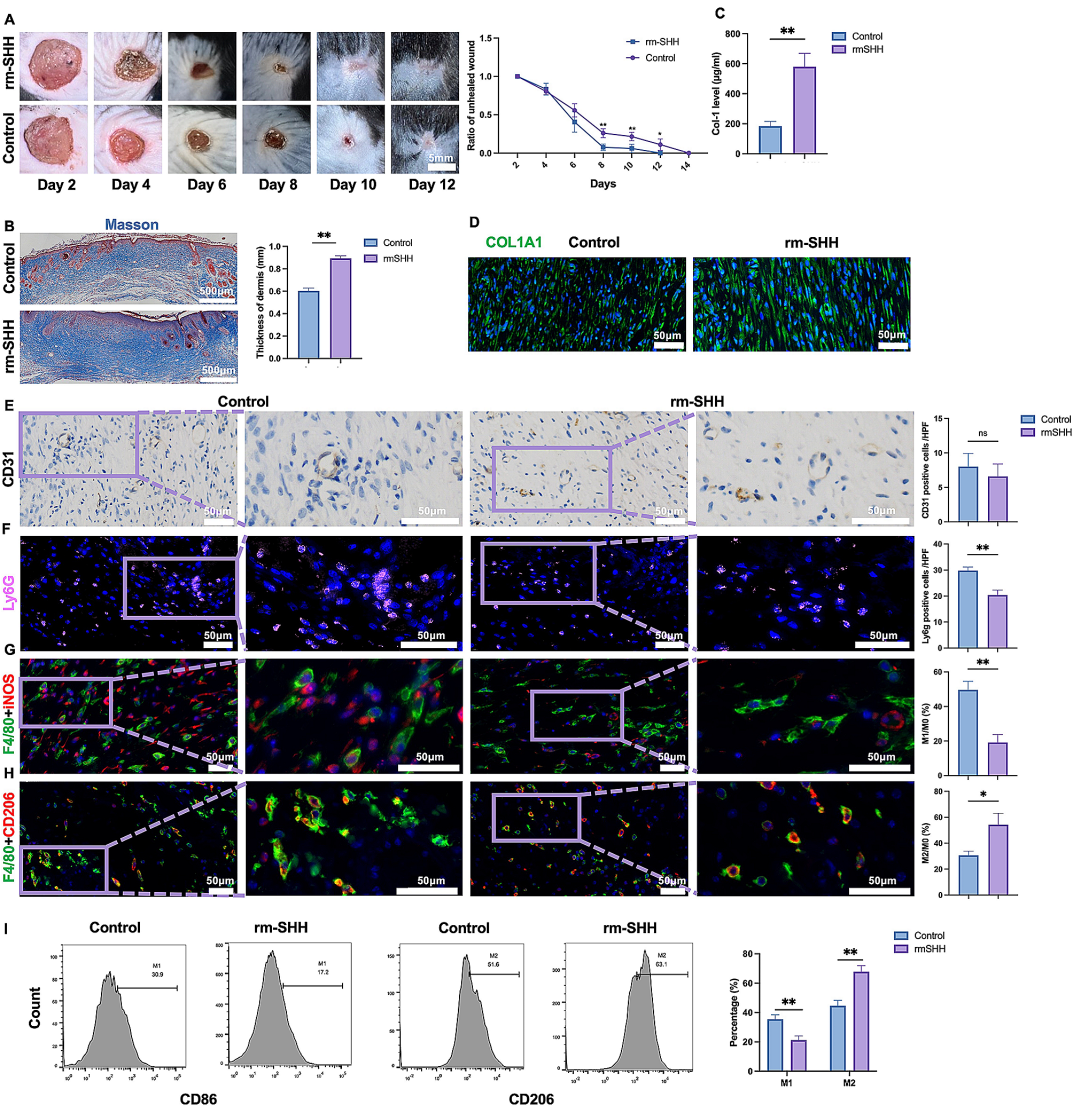
SHH promoted collagen deposition and polarization of M2 macrophage. **A**, wound model of dorsal skin was established in C57BL/6 mice, and the wound was treated with rmSHH or vector. The wound size during the healing period was recorded and measured (*n* = 6). **B**, masson staining was performed and the dermal layer were measured to evaluate the collagen deposition of the wound bed. **C**, ELISA was performed to measure the expression of type I collagen of the wound. **D**, IHC of CD31 was performed to compared the neovascularization of the two group. **E-G**, IF of Ly6G, F4/80, iNOS and CD206 were performed to evaluate the infiltration of neutrophil **(E)**, M1 **(F)** and M2 macrophage **(G)**. **H**, flow cytometry was performed to evaluate the macrophage phenotype of the wound bed. * *p* < 0.05, ** *p* < 0.01, *n* = 6

To explore the role of SHH during wound healing and scar formation, a mouse wound model was established at the back of mice, and rm-SHH was injected subcutaneously around the wound site. The administration of rm-SHH increased wound healing speed slightly (Fig. 2A). Collagen deposition is one of the main pathological characters of hypertrophic scar, thus the level of collagen deposition in mice was detected. Masson staining showed that rm-SHH stimulation increased the deposition of collagen, as well as the thickness of the dermis (Fig. 2B). IF and ELISA confirmed the increased expression of COL1A1 after rm-SHH stimulation (Fig. 2C-D). As we known that neovascularization and inflammatory response also play key roles in wound healing and fibrosis, CD31 (vessels), Ly6G (neutrophils), and F4/80 (macrophage) were detected. The IHC staining of CD31 showed that SHH injection had no influence on neovascularization of the wound (Fig. 2E). IF staining showed that the neutrophil was decreased, while the macrophage was not changed (Fig. 2F, Supplementary Fig. 1). Given the critical role of macrophage polarization in the process of wound healing and inflammatory response, the subtype of M1 and M2 macrophage was detected. Interestingly, M1 macrophage significantly decreased, while M2 macrophage increased (Fig. 2G, H, I). Thus, we speculated that SHH might induce the M2 polarization of macrophage from the wound site, which might be responsible for the collagen deposition.

### SHH signal enhanced macrophage efferocytosis and M2 polarization

**Fig. 3:**
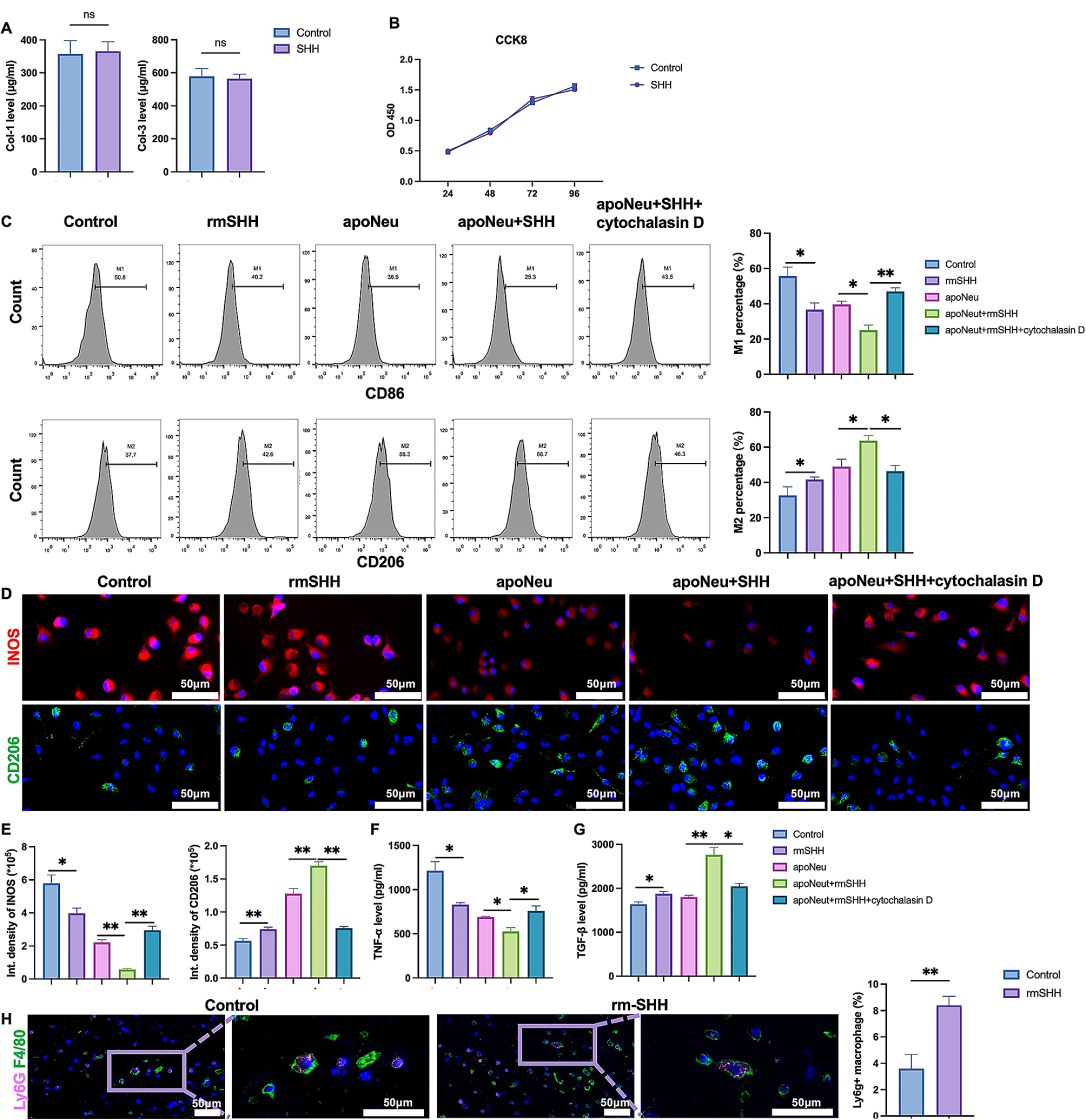
SHH signal enhanced macrophage efferocytosis and M2 polarization. **A-B**, fibroblasts were treated with rmSHH, and the ELISA and CCK8 were performed to evaluate the expression of type I/III collagen (**A**) and fibroblasts proliferation (**B**) (*n* = 3). **C**, Flow cytometry of macrophage were performed to evaluate the surface marker of M1 and M2 macrophage under SHH, apoptotic neutrophil (apoNeu), SHH + apoNeu, and SHH + apoNeu + cytochalasin D stimulation (*n* = 3). **D-E**, IF was performed to evaluate the expression of INOS and CD206 in macrophages under SHH, apoptotic neutrophil (apoNeu), SHH + apoNeu, and SHH + apoNeu + cytochalasin D stimulation (*n* = 6). **F-G**, ELISA was performed to evaluate the expression of TNF-α and TGF-β in macrophages under SHH, apoptotic neutrophil (apoNeu), SHH + apoNeu, and SHH + apoNeu + cytochalasin D stimulation (*n* = 3). **H**, IF of Ly6G and F4/80 were stained to show the colocalization of macrophage and neutrophil (*n* = 6). * *p* < 0.05, ** *p* < 0.01

To explore whether SHH has a direct effect on fibroblast, the main effector of skin wound healing and scar formation, we added the recombinant SHH into the medium of fibroblasts. The results showed that the direct activation of SHH signaling (indicated by the increased expression of Gli1, Supplementary Fig. 2) in fibroblasts from hypertrophic scar had no effect on the expression of COL1A1 and COL3A1 or the proliferation of fibroblast (Fig. 3A, B). Given the finding that SHH increased the M2 macrophage in cutaneous wound (Fig. 2G, H), we explored the direct effect of SHH on bone marrow derived macrophage, and found that rm-SHH stimulation significantly decreased M1 related markers such as CD86, INOS and TNF-α, while increased M2 related CD206 and TGF-β (Fig. 3C-G). During the IF experiment, we found partial neutrophil was colocalized with macrophage (Fig. 3H). As is known that macrophage is the main phagocyte during wound healing, and the aforementioned results that rm-SHH stimulation decreased neutrophils on wound bed, we wondered whether SHH stimulation wound enhance macrophage efferocytosis, one of the main procedures by which macrophages control inflammation and promote tissue repair [10]. After co-culture of macrophage and apoptotic neutrophil, we found SHH stimulation significantly increased M2 related markers and decreased M1 related markers, and these effects were eliminated by cytochalasin D, an inhibitor of macrophage phagocytic activity (Fig. 3C-F). As SHH showed no effect on neutrophils apoptosis (Supplementary Fig. 3), it might enhance the macrophage efferocytosis to regulate the macrophage polarization and aggravate cutaneous scar formation.

### SHH enhanced macrophage efferocytosis by promoting oxidative phosphorylation of macrophage

**Fig. 4:**
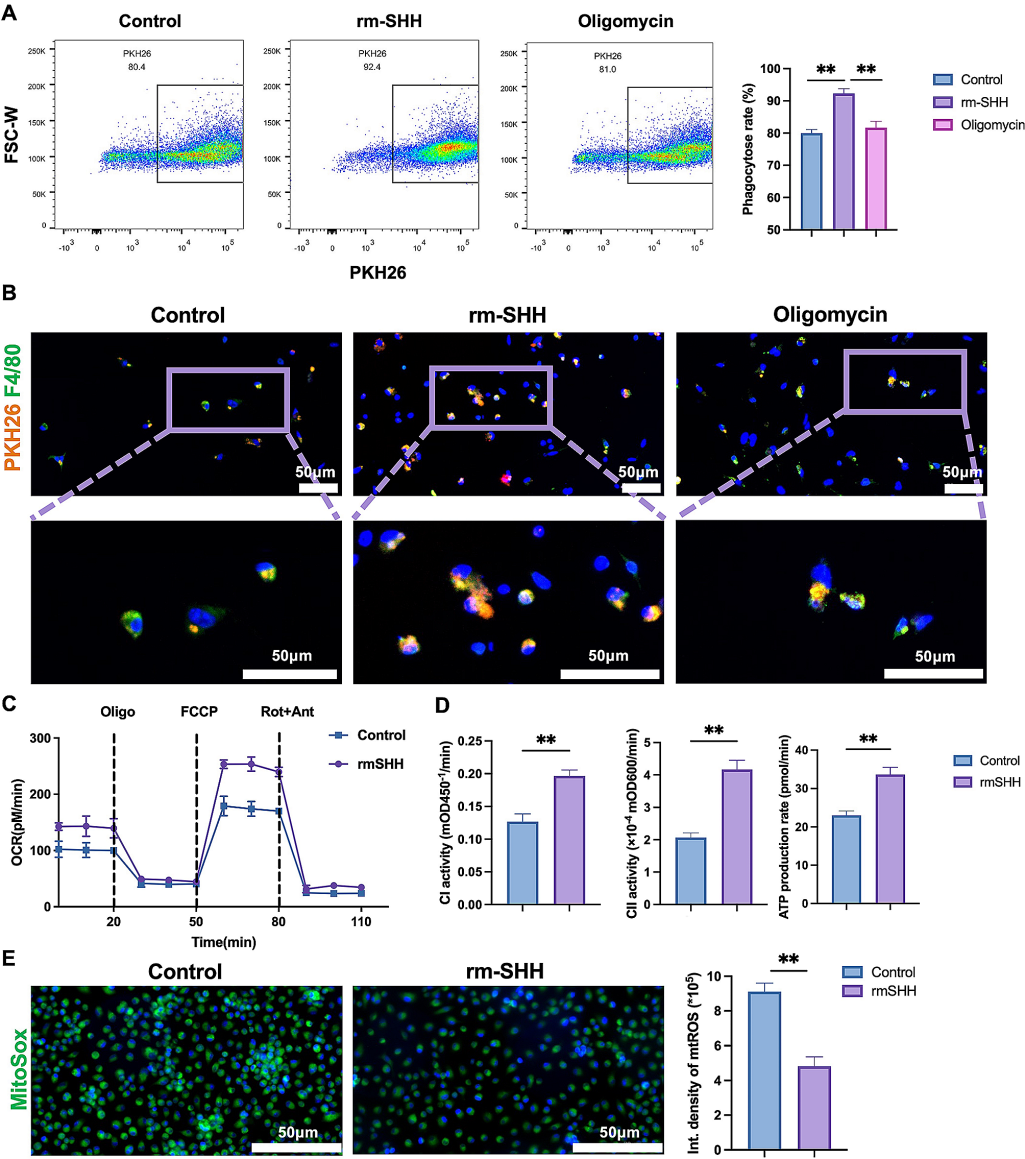
SHH enhanced macrophage efferocytosis by promoting oxidative phosphorylation of macrophage. **A-B**, apoptotic neutrophil was marked with PKH26 and incubated with bone marrow derived macrophage. Flow cytometry (**A**) and IF (**B**) were performed to evaluate the efferocytosis of macrophage, marked with PKH26 positive macrophage (*n* = 3). **C**, OCR and maximal respiration response of macrophage were measured with Seahorse (*n* = 3). **D**, the activity of complex I, II and ATP production were measured (*n* = 3). **E**, mtROS of macrophage was evaluated with MitoSOX™ Green(*n* = 6). * *p* < 0.05, ** *p* < 0.01

To verify the effect of SHH on efferocytosis, we marked apoptotic neutrophils with PKH26, and incubated with bone marrow derived macrophage. Flow cytometry and IF results showed that in SHH stimulation group, the percentage of PKH26 positive macrophage, which is the macrophage took in the apoptotic neutrophil carrying PKH26, was significantly increased (Fig. 4A, B). Efferocytosis is known to rely on ATP supply during engulfment and degradation activity [11, 12]. Because of these metabolic requirements, we wonder whether SHH administration affects the energy metabolism of macrophage. Thus, OCR was measured by Seahorse, and the results showed that SHH stimulation significantly increased basal OCR and maximal respiration response of macrophage (Fig. 4C). Meanwhile, we detected the activity of mitochondrial complex I and complex II. SHH stimulation significantly increased the activity of complex I and complex II, so did the ATP production (Fig. 4D). Meanwhile, the level of mtROS was significantly decreased with SHH stimulation (Fig. 4E). To verify the role of enhanced oxidative phosphorylation during efferocytosis, oligomycin was added into the culture medium. The results showed that the ratio of macrophage with phagocyted neutrophil was significantly decreased after inhibition of complex V (Fig. 4A, B). Therefore, SHH might enhance macrophage efferocytosis by promoting oxidative phosphorylation of macrophage.

### Vismodegib alleviated scar formation by targeting SHH signal

**Fig. 5:**
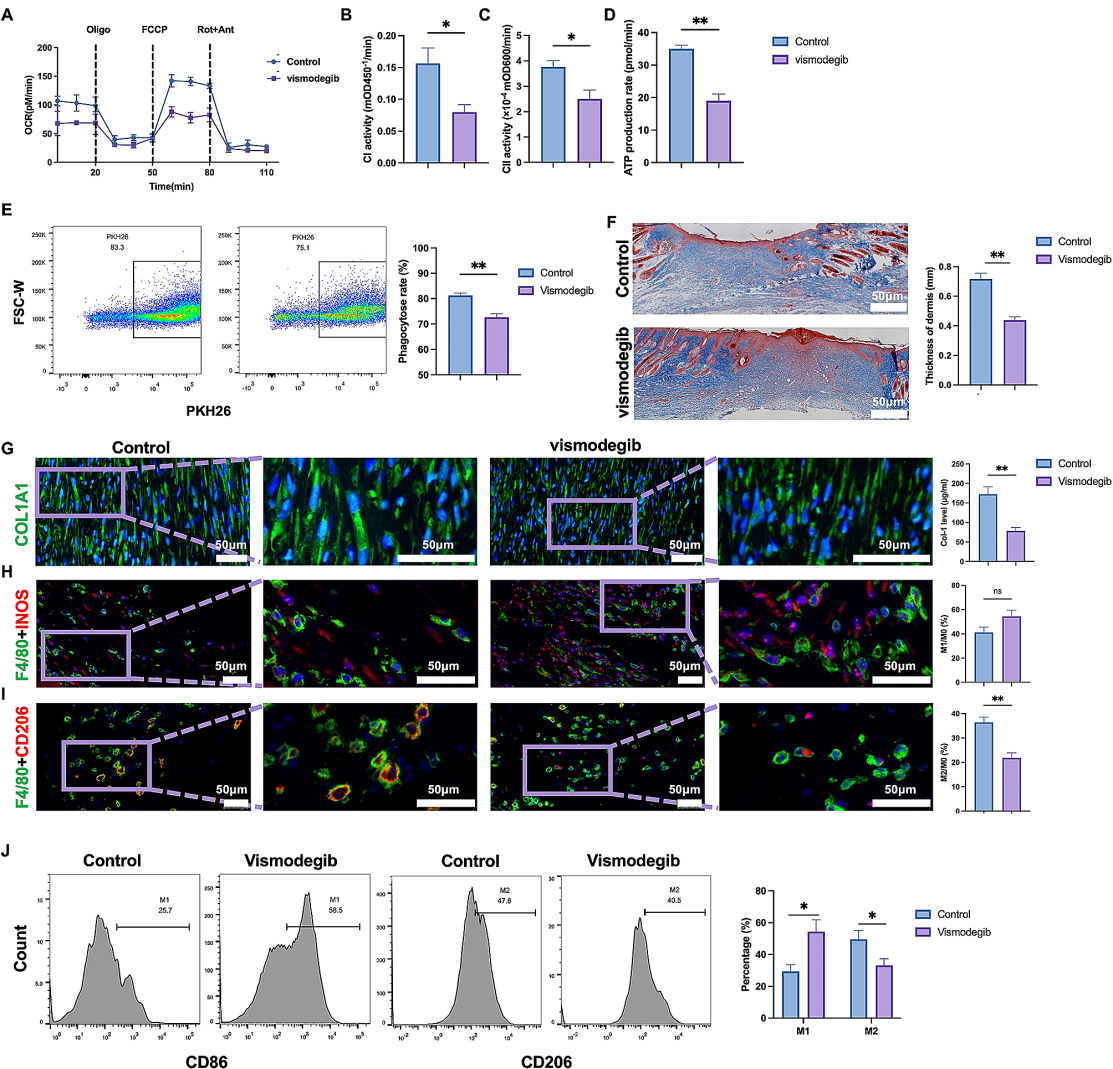
Vismodegib alleviated scar formation by targeting SHH signal. **A-D**, OCR, maximal respiration response, complex I, II and ATP production of macrophage treated with vismodegib were measured (*n* = 3). **E**, apoptotic neutrophil was marked with PKH26 and incubated with bone marrow derived macrophage. Flow cytometry was performed to evaluate the efferocytosis of macrophage treated with vismodegib (*n* = 3). **F-J**, wound model of dorsal skin was established in C57BL/6 mice, and vismodegib was administrated (*n* = 6). **F**, masson staining was performed and the dermal layer were measured to evaluate the collagen deposition of the wound bed. **G**, IF and ELISA was performed to evaluate the expression of type I collagen. **H-I**, IF of F4/80, iNOS and CD206 were performed to evaluate the macrophage infiltration of wound bed. **J**, flow cytometry was performed to evaluate the phenotype of macrophage from wound bed treated with vismodegib. * *p* < 0.05, ** *p* < 0.01

Given the important role of SHH on macrophage and its overexpression in hypertrophic scar, we wonder whether targeting SHH might alleviate scar formation by reversing efferocytosis and macrophage polarization. To simulate the in vivo microenvironment, we collected the supernatant of hypertrophic scar derived fibroblast and applied it as conditional medium for macrophage stimulation. In vitro experiments showed that during conditional medium culture, vismodegib, an inhibitor of SHH signal, significantly decreased basal OCR and maximal respiration response of macrophage, as well as the activity of complex I, complex II and ATP production of macrophage (Fig. 5A-D). Meanwhile, SHH inhibition significantly decreased the efferocytosis effect (Fig. 5E). At last, a mice model of dorsal skin wound was established to evaluate the potential role of vismodegib. Masson staining and IF staining showed that SHH inhibitor significantly alleviated the collagen deposition and the thickness of the dermis (Fig. 5F, G), and reduced M2 macrophage infiltration (Fig. 5H-J). Therefore, targeting SHH signaling might be a potential strategy for scar prevention.

## Discussion

Macrophages are critical mediators of tissue homeostasis, regulating tissue development and repair and defending against pathogens and debris. Macrophages are involved in the entire process of wound healing, including hemostasis, inflammation, proliferation and maturation [13]. It is well described that the prolonged activity of M2 macrophages is involved in scar formation, while the origin of M2 macrophages has led us to focus on the very beginning of wound healing, namely the inflammatory stage [14]. At this stage, the wound bed is flooded with apoptotic tissue cells and inflammatory cells, which are mainly removed by macrophages through phagocytosis, a process called efferocytosis [15]. First, macrophages sense “find me” signals released by apoptotic cells through surface-associated receptors such as the nucleotides ATP, UTP and the membrane lipid LysoPC. Macrophages then recognize “eat me” signals such as phosphatidylserine and calreticulin on the surface of apoptotic cells, triggering cytoskeletal rearrangement and the formation of phagosomes to engulf apoptotic cells. Finally, phagosomes fuse with lysosomes and degradation of apoptotic cells occurs [16]. This process is essential for tissue homeostasis and resolution of inflammation [15]. In addition, the activation of signals and changes in metabolites trigger the transition of macrophages from a pro-inflammatory M1 phenotype to an anti-inflammatory M2 phenotype [17]. This transition is characterized by increased secretion of anti-inflammatory cytokines, chemokines and repair factors that stimulate the activation and proliferation of myofibroblasts, which promote tissue repair and scar formation [6, 18]. In our study, we found that increased SHH promoted efferocytosis and M2 polarization, partly explaining the origin of increased M2 macrophages in scar formation.

In general, M1 macrophages rely mainly on glycolysis and show impaired tricarboxylic acid cycle and mitochondrial oxidative phosphorylation, whereas anti-inflammatory macrophages (M2) are more dependent on mitochondrial OXPHOS [19], which is the primary source of ATP in aerobic organisms [20]. As the process of efferocytosis requires large amounts of ATP [11], we investigated the macrophage energy system and found that SHH stimulation significantly increased macrophage OXPHOS, which provided the energy for cytoskeletal rearrangement, engulfment and digestion. This is consistent with a previous report that mitochondrial oxidative phosphorylation was enhanced and supported efferocytosis to treat atherosclerosis [11]. SHH has also been reported to promote oxidative phosphorylation in neurons and to protect neurons against a variety of stresses [21]. Further investigation revealed that SHH reduced mitochondrial fission and promoted mitochondrial elongation [21]. Indeed, mitochondrial dynamics, including fission and fusion, have been shown to play a critical role in the phenotype and function of cells [22]. For example, in macrophages, a recent study showed that disruption of optic atrophy 1 (OPA1), which controls mitochondrial fusion, correlated with excessive collagen deposition and impaired M1 commitment [23]. Another study found that inhibition of dynamin-related protein 1 (Drp1, which controls mitochondrial fission) significantly reduced macrophage phagocytosis of tumour cells [24]. In our study, we also evaluated mitochondrial fission and fusion, while the expression of OPA and DRP1 showed no difference with SHH stimulation (data not shown). Further studies are needed to explore the underlying mechanism.

Overall, our findings suggest that SHH-mediated efferocytosis and OXPHOS play critical roles in promoting tissue repair and scar formation. These results have important implications for the development of new therapies for wound healing and fibrosis. More research is needed to uncover the underline mechanism of SHH promoting OXPHOS.

### Electronic supplementary material

Below is the link to the electronic supplementary material.

Supplementary Figure 1. IF was performed to measure the infiltration of macrophage under SHH administration (n=6).

Supplementary Figure 2. RT-qPCR was performed to evaluate the mRNA level of Gli1 in fibroblasts under SHH administration (n=3). ** p<0.01.

Supplementary Figure 3. Flow cytometry was performed to evaluate the apoptotic rate of neutrophil with SHH stimulation (n=3).

## Data Availability

All data generated or analyzed during this study are available from the corresponding author on reasonable request.
