# Supplementary figures and images for: SHH induces macrophage oxidative phosphorylation and efferocytosis to promote scar formation
Source: Cell Commun Signal. 2024 Jun 19;22:336. doi: 10.1186/s12964-024-01692-w (PMC11186229; doi:10.1186/s12964-024-01692-w)

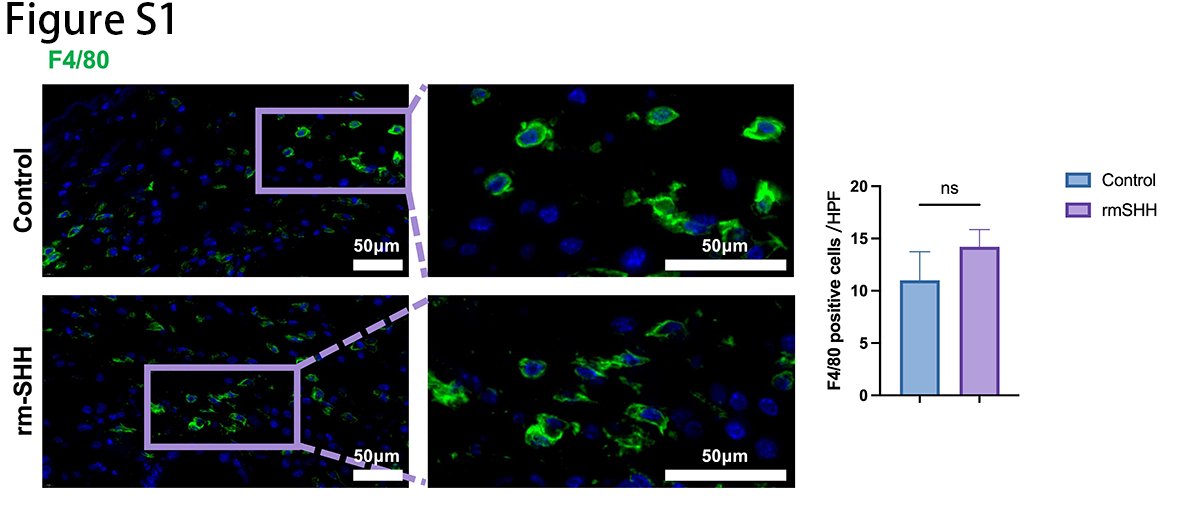

Supplement: Supplementary file 1 — Supplementary Figure 1. IF was performed to measure the infiltration of macrophage under SHH administration (n=6). [file 12964_2024_1692_MOESM1_ESM.png]

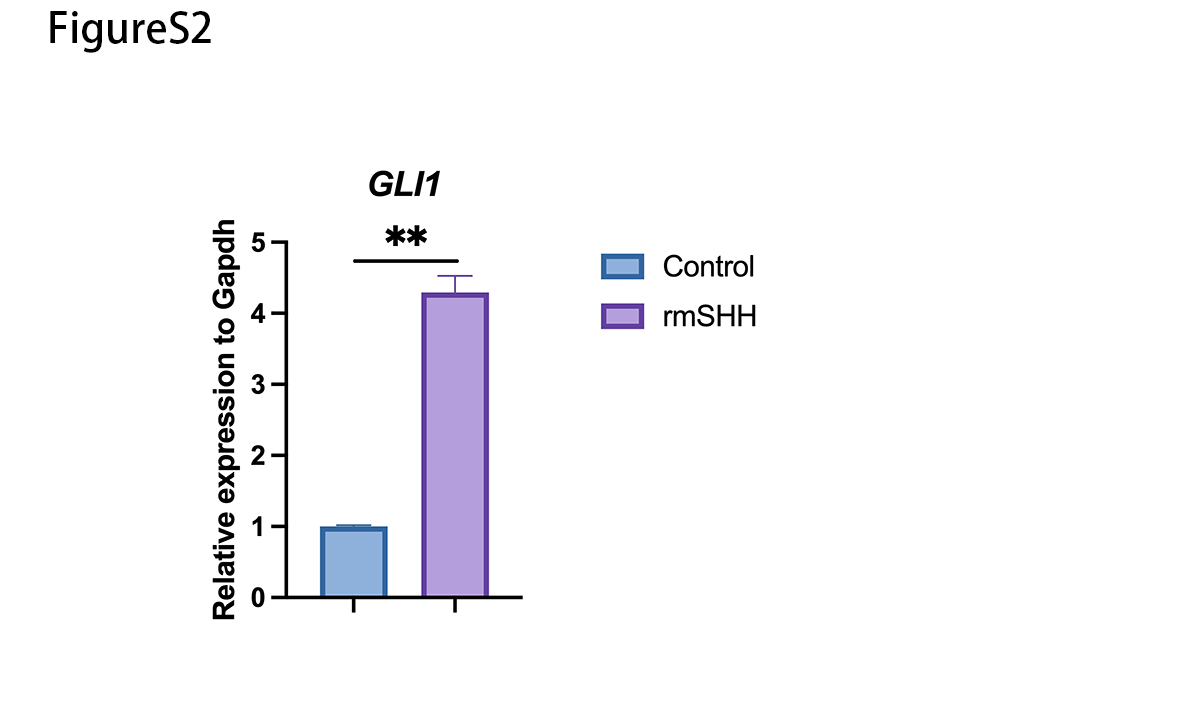

Supplement: Supplementary file 2 — Supplementary Figure 2. RT-qPCR was performed to evaluate the mRNA level of Gli1 in fibroblasts under SHH administration (n=3). ** p<0.01. [file 12964_2024_1692_MOESM2_ESM.png]

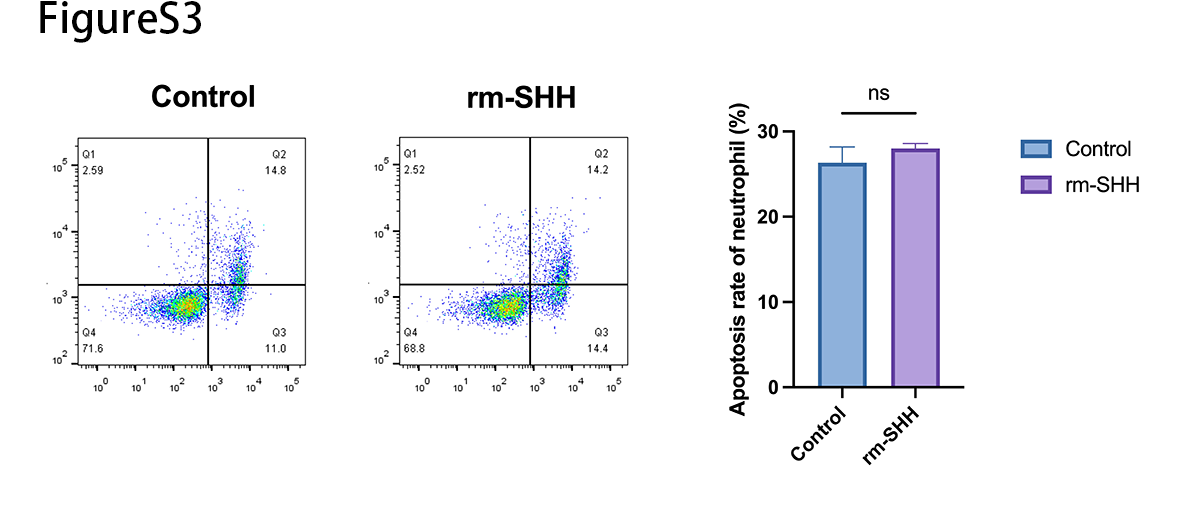

Supplement: Supplementary file 3 — Supplementary Figure 3. Flow cytometry was performed to evaluate the apoptotic rate of neutrophil with SHH stimulation (n=3). [file 12964_2024_1692_MOESM3_ESM.png]
